# Early detection and genetic characterization of clade 2.3.4.4b H5N1 and H5N9 highly pathogenic avian influenza viruses at the onset of fall migration in wild birds during october 2025 in South Korea

**DOI:** 10.3389/fcimb.2026.1755375

**Published:** 2026-01-23

**Authors:** Young-Jae Si, Sun-Hak Lee, Ha-Eun Lee, Dong-Ju Kim, Hyesung Jeong, Suwoong Lee, Dong-Hun Lee

**Affiliations:** 1 Wildlife Disease Research Team, National Institute of Wildlife Disease Control and Prevention, Gwangju, Republic of Korea; 2 Wildlife Health Laboratory, College of Veterinary Medicine, Konkuk University, Seoul, Republic of Korea; 3 Avian Disease Laboratory, College of Veterinary Medicine, Konkuk University, Seoul, Republic of Korea

**Keywords:** clade 2.3.4.4b, Common teal, H5N1, H5N9, highly pathogenicity avian influenza, phylogenetic analysis, South Korea

## Abstract

Highly pathogenic avian influenza (HPAI) viruses of clade 2.3.4.4b continue to diversify through reassortment with co-circulating low-pathogenic avian influenza (LPAI) viruses and are repeatedly introduced into South Korea via migratory flyways. During national wild bird surveillance in October 2025, two HPAI viruses of different subtypes, H5N1 and H5N9, were detected in Common teals in the southwestern Korea. Whole-genome sequencing confirmed both isolates as clade 2.3.4.4b viruses belonging to the G2c sub-lineage. Phylogenetic analysis showed that the H5N1 virus possessed a genomic backbone related to the 22G4 genotype circulating in Korea during the 2022–2023 season, incorporating a PB1 segment derived from LPAI viruses. The H5N9 virus represented a distinct reassortant carrying an NA gene closely related to H11N9 LPAI viruses and internal segments associated with KorD and KorC genotypes prevalent in the same season. Bayesian time-scaled analysis indicated that both isolates originated from an East Asian H5Nx lineage with a common ancestor around 2019, and that the H5N1 virus diverged from a closely related Chinese strain in late 2023. Both viruses harbored multiple mammalian-adaptation markers, including substitutions commonly detected in recent East Asian HPAI strains. These findings demonstrate ongoing inter-lineage reassortment between regional HPAI and LPAI gene pools, emphasizing the continued role of migratory waterfowl in introducing emerging variants into Korea. The early-season detection of genetically distinct reassortants highlights the importance of sustained wild bird surveillance, rapid genomic characterization, and international data sharing to track the evolution and spread of newly emerging HPAI lineages.

## 1 Introduction

Highly pathogenic avian influenza (HPAI) subtype H5Nx viruses of the A/Goose/Guangdong/1/1996 (Gs/Gd) lineage continue to pose a major threat to poultry production and global public health (Alexander, 1996; Gashaw, 2020). Since their emergence, Gs/Gd-lineage H5 viruses have diversified into ten primary clades (0–9) and numerous subclades, frequently reassorting with other influenza A viruses (Neumann et al., 2010; Bertran et al., 2017; Cui et al., 2022). The clade 2.3.4.4b H5N1 viruses have evolved into a panzootic lineage—following emergence in 2020, they have now established a global presence across all continents and expanded their host range into a wide array of mammalian species, including dairy cattle in 2024 and marine mammals in 2023–2025, raising heightened zoonotic and One Health concerns (Li et al., 2024; Chrzastek et al., 2025; Mena Vasquez et al., 2025).

Since 2020, clade 2.3.4.4b H5Nx viruses have caused annual winter epidemics in South Korea, introduced via migratory waterfowl along the East Asian–Australasian flyway (Lee et al., 2025). While H5N8 predominated during the 2020–2021 season (Baek et al., 2021; Si et al., 2024), H5N1 caused outbreaks in both the 2021–2022 and 2022–2023 seasons (Cha et al., 2023; Kang et al., 2023; Seo et al., 2024a). The 2023–2024 season featured concurrent H5N1 and H5N6 circulation, with novel reassortants (Kang et al., 2023; Cho et al., 2024b; Cho et al., 2024a; Seo et al., 2025). During the 2024–2025 winter season in South Korea, surveillance of HPAI viruses revealed incursions of clade 2.3.4.4b H5N1 viruses via wild waterfowl and the first mammalian spillover to a leopard cat in March 2025 (Si et al., 2025a; Si et al., 2025b). Between October 2024 and March 2025, under the national surveillance programs, a total of 53 and 34 HPAI cases were confirmed in wild birds (http://wadis.go.kr) and poultry farms (home.kahis.go.kr), respectively. For early detection of HPAI virus in wild birds, the National Institute of Wildlife Disease Control and Prevention (NIWDC), Korea has conducted active surveillance of wild birds through intensified sampling at high-risk migratory habitats during fall migration.

Here, we report the early detection of two clade 2.3.4.4b HPAI viruses H5N1 and H5N9, identified at the onset of fall migration of wild waterfowl in 2025. The viruses were detected in fecal and oropharyngeal samples from wild Common teals (*Anas crecca*) in Jeollabuk-do, South Korea. Whole-genome sequencing was performed, and the complete genomes were deposited in the GISAID database. Comparative phylogenetic analyses were conducted to determine their genetic characteristics and evolutionary origins.

## 2 Material and methods

### 2.1 Isolation and genome sequencing of viruses

On 27 October 2025, as a part of the national wild bird surveillance program, a total of 66 fresh fecal samples from wild waterfowl were collected along the lower reaches of the Mangyeong River in Gunsan, Jeollabuk-do, South Korea (GPS: 35°55′41.4″ N, 126°42′52.8″ E). On 28 October 2025, active capture of wild waterfowl was carried out along the Gobu Stream in Buan, Jeollabuk-do (GPS: 35°41′02.5″ N, 126°44′15.3″ E). Oropharyngeal and cloacal swabs were collected individually using sterile polyester-tipped applicators and placed in cryovials containing phosphate-buffered saline (PBS) supplemented with 400 µg/mL gentamicin.

Both fecal and swab samples were homogenized by vortexing for 1 min, followed by centrifugation at 3,000 rpm for 10 min. Clarified fecal supernatants were pooled into 13 groups (5–6 specimens per pool) for preliminary virus screening, after which samples from positive pools were tested individually. For swab samples, oropharyngeal and cloacal supernatants collected from the same bird were pooled per bird. For virus isolation, the supernatants were filtered through 0.45-µm Minisart syringe filters (Sartorius, Göttingen, Germany) and inoculated into 10-day-old specific-pathogen-free (SPF) embryonated chicken eggs. Eggs were incubated at 37 °C for 72 h, and allantoic fluids were harvested and examined for hemagglutination (HA) activity using 0.5% chicken red blood cells. For host species identification and confirmation of virus infection at the individual level, each fecal specimen was further processed using the same screening workflow. Host species for the viruses were identified by DNA barcoding as previously described (Lee et al., 2010).

For swab and HA-positive allantoic fluid samples, viral RNA was extracted from the allantoic fluid using the Maxwell RSC Simply RNA Tissue Kit (Promega, Madison, WI, USA) according to the manufacturer’s protocol. Extracted RNA was screened for the matrix (M) and H5 genes of avian influenza virus by real-time reverse transcription PCR (rRT-PCR) following standard procedures (Spackman, 2020), and HA/NA subtyping was performed using the LiliF AIV Multi-tube RT-PCR Kit (iNtRON Biotechnology, Korea).

### 2.2 Whole genome sequencing and sequence analysis

To conduct a full genome analysis, complementary DNA of positive samples for the rRT-PCR was synthesized with the SuperScript III First-Strand Synthesis System (Invitrogen, Carlsbad, CA, USA), and all eight viral gene segments were amplified using AccuPrime Pfx DNA Polymerase (Invitrogen) as previously described (Lee, 2020). Indexed sequencing libraries were prepared with the Nextera DNA Flex Library Prep Kit (Illumina, San Diego, CA, USA) and sequenced on an Illumina MiSeq platform using a 150-bp paired-end strategy. Raw reads were quality-filtered and trimmed with BBDuk v38.84 (minimum Q = 30), and *de novo* assembly was performed with SPAdes v3.15.5 (Bankevich et al., 2012; Whitham, 2020). Trimmed reads were then mapped to the assembled contigs using Minimap v2.24 (Li, 2018) and visualized in Geneious Prime (Kearse et al., 2012). Consensus genome sequences were generated through reference-guided assembly and deposited in the GISAID EpiFlu database (accession IDs EPI_ISL_20248219 and EPI_ISL_20248220). Clade classification of H5 sequences was conducted using the online subspecies classification tool available at BV-BRC (https://www.bvbrc.org/app/SubspeciesClassification). Molecular markers of mammalian adaptation, pathogenicity, and drug resistance were identified using the FluMut tool (Giussani et al., 2025). In the FluMut analysis, in addition to the sequences isolated in this study, eight clade 2.3.4.4b HPAI viruses that were phylogenetically closely related to the present isolates were also included. These comprised seven H5N1 viruses— one duck isolate from China in 2024 (A/duck/Guizhou/S1711/2024) (Shi et al., 2025), one duck isolate from Vietnam in 2023 (A/duck/Vietnam/Raho4-Cd-23-1744/2023), one duck isolate from Japan in 2024 (A/duck/Tottori/NK11F5-8/2024), two from the 2022–2023 outbreaks in South Korea (A/chicken/Korea/H587/2022, A/Em/Korea/22WF182-9P/2022) (Seo et al., 2024a; Cha et al., 2025), and one leopard cat isolate from South Korea in 2025 (A/Leopard_cat/Korea/24WM130/2024) (Si et al., 2025b)—as well as one H5N6 virus isolated from a human case in China in 2024 (A/HeFei04171/2024) (Sun et al., 2025).

### 2.3 Phylogenetic analysis

Consensus genome sequences were analyzed using the GISAID BLAST query function (https://www.gisaid.org/). From the top 500 hits, sequences with high identity, ranging from 99.8 to 100% depending on the segment, were removed with ElimDupes (https://www.hiv.lanl.gov/content/sequence/elimdupesv2/elimdupes.html). In addition to the general dataset, gene-specific datasets were curated to improve phylogenetic resolution. For the HA gene, G2a and G2d sub-lineage sequences were included to refine outgroup placement and lineage classification. For the NA gene, recent Korean seasonal H5N1 HPAI strains were added to assess temporal relatedness. For the MP gene, H5Nx viruses circulating from 2006 to 2025 were compiled, and highly similar taxa were removed using ElimDupes to reduce redundancy. Multiple-sequence alignment was performed using MAFFT (Katoh and Standley, 2013), and maximum-likelihood phylogenetic trees for each gene were inferred in RAxML v8.0 under the general time-reversible (GTR) substitution model with gamma-distributed rate variation and 1,000 bootstrap replicates (Stamatakis, 2014). The resulting trees were visualized with iTOL, and clusters were defined as distinct when bootstrap support exceeded 70 and nucleotide sequence identity exceeded 98% (Letunic and Bork, 2021).

A time-scaled phylogeny of the HA gene was reconstructed in BEAST v1.10.4 using the Hasegawa–Kishino–Yano substitution model and an uncorrelated log-normal relaxed clock, with a Gaussian Markov Random Field (GMRF) Bayesian Skyride coalescent prior (Baele et al., 2025). Three independent Markov Chain Monte Carlo (MCMC) chains were run for 50 million iterations each, with the first 10% discarded as burn-in. All estimated parameters achieved effective sample sizes > 200 and were examined using TRACER v1.5 (Rambaut et al., 2018). The maximum clade credibility (MCC) tree was generated in TreeAnnotator and visualized with FigTree v1.4.4 (http://tree.bio.ed.ac.uk/software/figtree/). The time to the most recent common ancestor (tMRCA) was estimated from node height distributions in the MCC tree.

## 3 Results

### 3.1 Virus isolation and identification

From 66 fecal samples collected along the Mangyeong River on 27 October 2025, 13 pooled samples were prepared for initial screening, of which 10 samples tested positive for influenza A virus. Subsequent egg inoculation of each fecal specimen yielded one H5N1 HPAI virus, along with several low-pathogenic avian influenza (LPAI) viruses, including three H6N1, two H6N6, and one H5N3 isolates. DNA barcoding of the HPAI-positive specimen identified the host species as Common teal (*Anas crecca*).

On 29 October 2025, 16 wild waterfowl were captured along the Gobu Stream in Buan, comprising twelve mandarin ducks (*Aix galericulata*), one mallard (*Anas plantyrhychos*), and three common teals. rRT-PCR screening of pooled oropharyngeal and cloacal swabs revealed one common teal positive for the M and H5 genes, with cycle threshold (Ct) values of 26.98 and 26.07, respectively. Virus isolation in embryonated chicken eggs resulted in HA-positive allantoic fluid, which was further confirmed as positive by rRT-PCR for the M and H5 genes. We successfully isolated two clade 2.3.4.4b H5Nx HPAI viruses, namely an H5N1 subtype, A/Common_teal/Korea/25WF154-8P/2025 (hereafter 25WF154-8P), and an H5N9 subtype, A/Common_teal/Korea/25WS011-14/2025 (hereafter 25WS011-14).

### 3.2 Whole-genome sequencing and sequence analysis

Next-generation sequencing (NGS) of the isolated viruses, 25WF154-8P and 25WS011-14, generated 91,189 and 151,560 read counts, respectively, allowing complete coding sequences to be assembled for all eight gene segments. The mean sequencing depth across all segments exceeded 60×, supporting high-confidence consensus genome reconstruction. The isolates were confirmed as HPAI viruses based on the presence of multiple basic amino acids at the HA cleavage site (PLREKRRKR/G) and classified as an H5 clade 2.3.4.4b (Lee et al., 2024).

Mutation analysis revealed that the two isolates obtained in this study possessed numerous mammalian-adaptation markers, with 25WF154-8P and 25WS011–14 harboring 29 and 28 substitutions, respectively (**Table 1**). In the HA gene, substitutions associated with increased virulence in mice (S107R, T108I, T134A) and enhanced binding to α2-6–linked sialic acid receptors (T156A, V182N, K218Q, S223R) were identified (Suttie et al., 2019). The N154D substitution, which has been implicated in enhanced replication and increased polymerase activity in mammalian cells, was also present. Internal gene segments carried multiple substitutions previously linked to increased or enhanced virulence in mice, including M1 (N30D, I43M, T215A), NS1 (P42S, L103F, I106M), PB1 (D622G), and a series of polymerase-complex mutations in PB2 (L89V, G309D, R477G, I495V, V598T), as well as PB1-F2 N66S (Suttie et al., 2019). Additional substitutions associated with enhanced replication or increased polymerase activity in mammalian cells were also detected, including NS1 (K55E, K66E, I106M, C138F), PB2 (K389R, V598T), PB1 (D3V), and PA (N409S) (Suttie et al., 2019). Notably, none of the isolates possessed the well-known mammalian-adaptation markers PB2-E627K or PB2-D701N (Zhu et al., 2015). Mutations identified in viruses associated with zoonotic infections—such as PB2-K699R in the human H5N6 isolate from China and NS-D74N in the H5N1 virus from a Korean leopard cat—were absent from all poultry- and wild-bird-derived HPAI viruses examined in this study, including our isolates (Suttie et al., 2019).

**Table 1:** Comparison of mammalian adaptation markers among Korean wild birds isolates in the 2025–2026 winter season, genetically related clade 2.3.4.4b highly pathogenic H5Nx viruses and the Korean leopard cat isolate.

Isolate	subtype	PB2										PB1		PB1-F2	PA	HA	
		L89V^a^	I292V^a^	G309D^a^	T339K^a^	K389R^b^	R477G^a^	I495V^a^	V598T^ab^	A676T^a^	K699R^cd^	D3V^b^	D622G^ae^	N66S^df^	N409S^b^	S107R^a^	T108I^a^
A/duck/Guizhou/ S1711/2024	H5N1	V	V	D	K	R	G	V	T	T	K	V	G	S	S	R	I
A/duck/Vietnam/ Raho4-Cd-23-1744/2023	H5N1	V	V	D	K	R	G	V	T	T	K	V	G	S	S	R	I
A/duck/Tottori/ NK11F5-8/2024	H5N1	V	I	D	K	R	G	V	T	A	K	V	G	S	S	R	I
A/chicken/Korea/ H587/2022	H5N1	V	I	D	K	R	G	V	T	T	K	V	G	S	S	R	I
A/Em/Korea/ 22WF182-9P/2022	H5N1	V	V	D	K	R	G	V	T	A	K	V	G	S	S	R	I
A/leopard cat/Korea/ 24WM130/2024	H5N1	V	V	D	K	R	G	V	T	T	K	V	G	S	S	R	I
A/HeFei04171/2024	H5N6	V	I	D	K	R	G	V	T	T	R	V	G	S	S	R	I
A/Common teal/Korea/ 25WF154-8P/2025	H5N1	V	V	D	E	R	G	V	T	T	K	V	G	S	S	R	I
A/Common teal/Korea/ 25WS011-14/2025	H5N1	V	I	D	K	R	G	V	T	A	K	V	G	S	S	R	I
Isolate	subtype	HA						M1			NS-1						
		T134A^g^	N154D^hi^	T156A^jk^	V182N^k^	K218Q^k^	S223R^k^	N30D^a^	I43M^a^	T215A^a^	P42S^a^	K55E^hi^	K66E^hi^	D74N^a^	L103F^a^	I106M^c^	C138F^chi^
A/duck/Guizhou/ S1711/2024	H5N1	A	D	A	N	Q	R	D	M	A	S	E	E	D	F	M	F
A/duck/Vietnam/ Raho4-Cd-23-1744/2023	H5N1	A	N	A	N	Q	R	D	M	A	S	E	E	D	F	M	F
A/duck/Tottori/ NK11F5-8/2024	H5N1	A	D	A	N	Q	R	D	M	A	S	E	E	D	F	M	F
A/chicken/Korea/ H587/2022	H5N1	A	D	A	N	Q	R	D	M	A	S	E	E	D	F	M	F
A/Em/Korea/ 22WF182-9P/2022	H5N1	A	D	A	N	Q	R	D	M	A	S	E	E	D	F	M	F
A/leopard cat/Korea/ 24WM130/2024	H5N1	A	N	A	N	Q	R	D	M	A	S	E	E	N	F	M	F
A/HeFei04171/2024	H5N6	A	D	A	N	Q	R	D	M	A	S	E	E	D	F	M	F
A/Common teal/Korea/ 25WF154-8P/2025	H5N1	A	D	A	N	Q	R	D	M	A	S	E	E	D	F	M	F
A/Common teal/Korea/ 25WS011-14/2025	H5N1	A	D	A	N	Q	R	D	M	A	S	E	E	D	F	M	F

a Indicates substitutions linked to increased virulence in mice.

b Indicates substitutions associated with increased replication in mammalian cells.

c Indicates substitutions associated with increased viral replication in mammalian cells.

d Indicates substitutions linked to enhanced virulence in mice.

e Indicates substitutions linked to increased polymerase activity in mice.

f Indicates substitutions reported to enhance viral replication in mice.

g Indicates substitutions associated with increased viral replication in mice lung tissue.

h Indicates substitutions reported to enhance replication in mammalian cells.

i Indicates substitutions associated with increased polymerase activity in mammalian cells.

j Indicates substitutions associated with increased transmission in guinea pigs.

k Indicates substitutions shown to increase binding affinity for α2-6–linked sialic acid receptors.

### 3.3 Phylogenetic analysis

**Figure 1:**
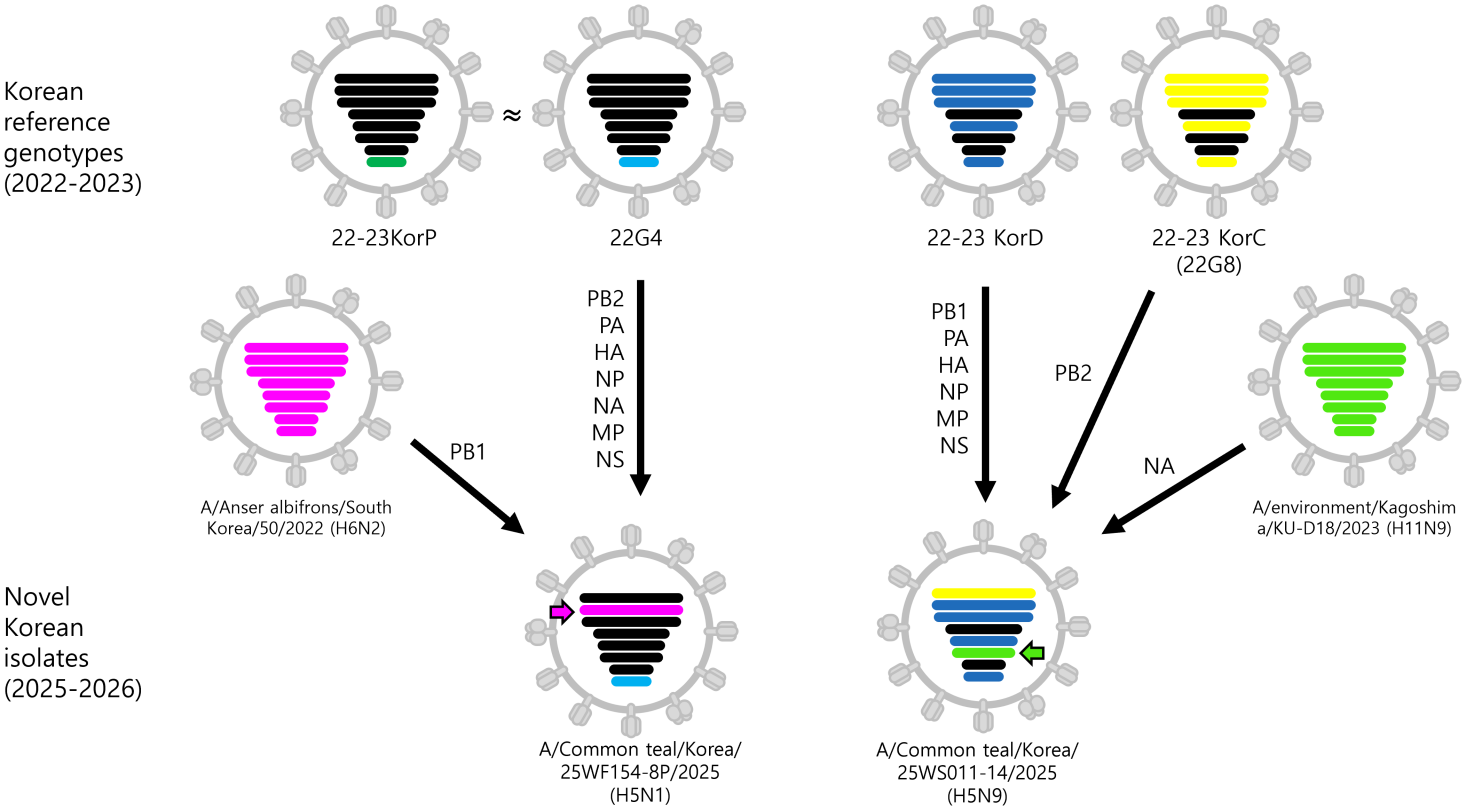
Schematic representation of the genotypes of H5N1 and H5N9 highly pathogenic avian influenza (HPAI) viruses isolated in South Korea in October 2025. Bars represent eight gene segments of the avian influenza virus in the following order (top to bottom): polymerase basic 2 (PB2), polymerase basic 1 (PB1), polymerase acidic (PA), hemagglutinin (HA), nucleoprotein (NP), neuraminidase (NA), matrix (M), and non-structural (NS). Different bar colors indicate different virus origins estimated from maximum-likelihood phylogenetic trees. Genotypes detected during the 2022–2023 season are shown for comparative reference.

Phylogenetic trees constructed from the eight gene segments revealed that the HA genes of the H5N1 isolate (25WF154-8P) belonged to the G2c sub-lineage of clade 2.3.4.4b (**Supplementary Figure 1**) (Nabeshima et al., 2023). The PB2, PA, NP, NA, and MP genes clustered with HPAI viruses detected in poultry from Korea (2022–2023), Vietnam (2023), and China (2024), showing close relatedness to the 22G4 (KorP) genotype identified in domestic poultry during the 2022–2023 winter season (Seo et al., 2024b; Cha et al., 2025). The NS gene displayed a similar topology, grouping with the same 22G4 lineage viruses. In contrast, the PB1 gene grouped with LPAI viruses isolated from wild birds in Korea in 2022. These findings indicate that the 25WF154-8P virus likely originated from a reassortant derived from the 22G4 genotype constellation, incorporating a PB1 segment from an LPAI progenitor (**Figure 1**).

Similarly, the H5N9 isolate (25WS011-14) also possessed an HA gene within the G2c sub-lineage but exhibited a distinct internal-gene constellation (**Supplementary Figure 1**). All segments except PB2 and NA clustered with the KorD genotype viruses from Korean poultry in 2022–2023, while PB2 was most closely related to the 22G8 (KorC) genotype (Seo et al., 2024b; Cha et al., 2025). However, NA grouped with H11N9 LPAI viruses detected in Korea, Japan, and Russia in 2023. Together, these findings indicate that 25WS011–14 exhibits a mosaic internal-gene constellation derived predominantly from KorD genotype viruses, incorporating a PB2 segment from KorC genotype viruses and a NA segment from contemporary H11N9 LPAI lineages (**Figure 1**).

**Figure 2:**
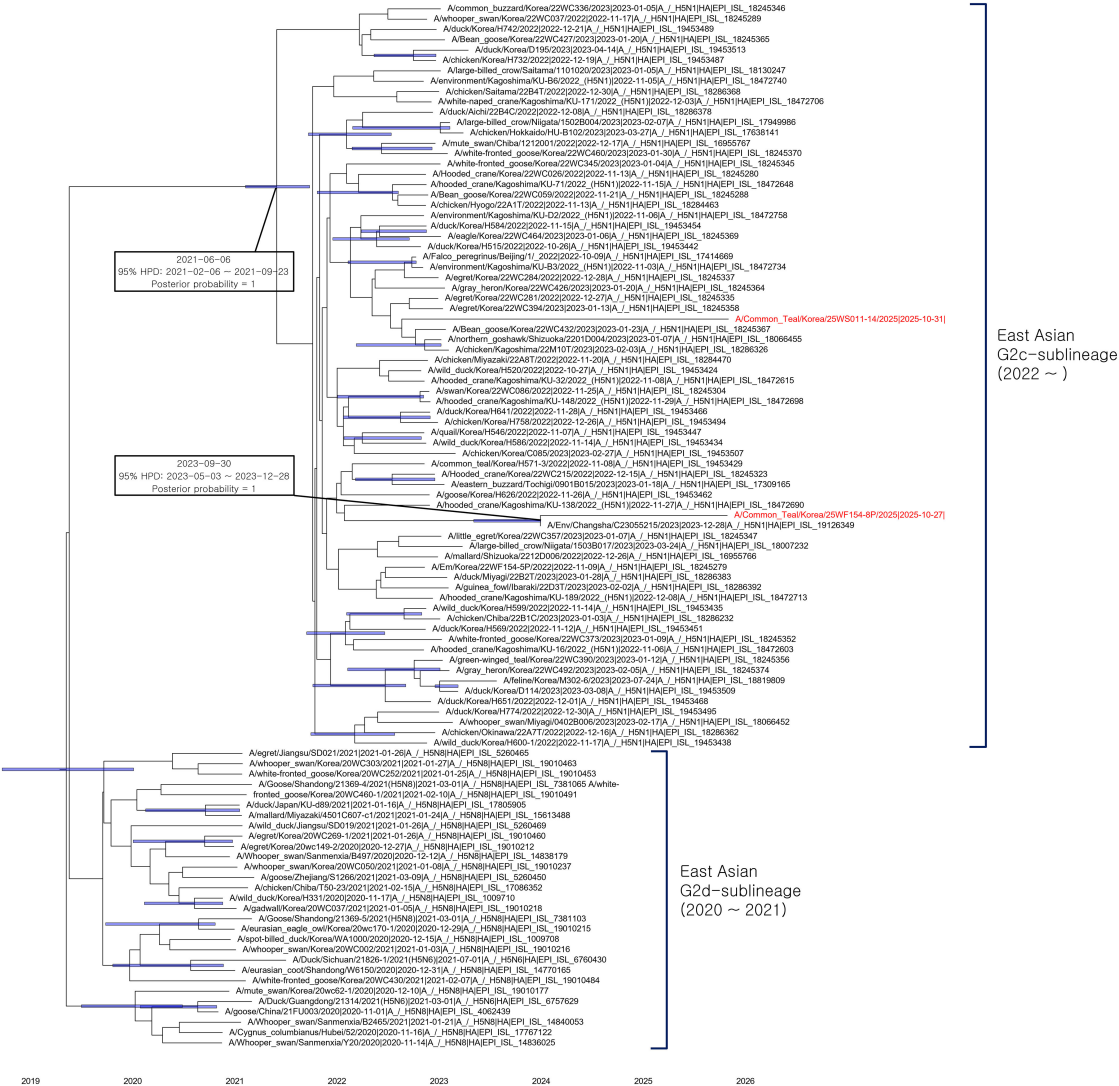
Time-scaled Maximum clade credibility (MCC) tree constructed using the hemagglutinin (HA) gene of G2c and G2d sublineages of H5 highly pathogenic avian influenza (HPAI) viruses. Sequences were obtained from the GISAID database, and the H5N1 and H5N9 viruses isolated in South Korea in October 2025 are highlighted in red. Node bars represent the 95% highest posterior density (HPD) intervals of estimated node heights with a posterior probability (PP) > 0.5. The horizontal axis represents time expressed in decimal years. Black-outlined boxes mark two selected nodes, summarizing their estimated time to the most recent common ancestor (tMRCA), associated 95% HPD intervals, and PP values.

Based on the Bayesian phylogeny of the HA gene, the index H5N1 and H5N9 viruses did not cluster immediately adjacent to one another in phylogenies but belonged to the G2c subgroup of clade 2.3.4.4b, which has been detected in East Asia since 2022 (**Figure 2**). This topology indicates two independent introductions into South Korea most likely via wild birds. The tMRCA of the G2c clade was estimated at 2021-06-06 (95% HPD interval: 2021-02–06 to 2021-09-23), with a posterior probability of 1. These results suggest that clade 2.3.4.4b viruses have been continuously maintained and circulating in wild bird populations in East Asia since mid-2021, with repeated spillovers from wild bird populations causing outbreaks in poultry and occasional infections in wild mammals.

The 25WF154-8P virus formed a monophyletic clade with A/Env/Changsha/C23055215/2023(H5N1) from China with an estimated tMRCA of 30 September 2023 (95% HPD interval: 3 May–28 December 2023). The long branch separating these two sequences from their common ancestry reflects the scarcity of genomic surveillance data from East Asia. This temporal and phylogenetic pattern indicates that these viruses share a common ancestor that diverged in late 2023, after which they circulated undetected and evolved independently in separate transmission chains.

## 4 Discussion

Continued reassortment of clade 2.3.4.4b H5 HPAI viruses with co-circulating LPAI viruses has generated a diverse genetic pool that has disseminated globally (Cui et al., 2022; Li et al., 2024; Chrzastek et al., 2025; Mena Vasquez et al., 2025). ML phylogenies revealed that the novel reassortant H5N1 virus, 25WF154-8P, detected in 2025 contained seven gene segments closely related to HPAI viruses from the 2022–2023 outbreak in South Korea, with the PB1 segment derived from LPAI virus detected in the East Asian–Australasian flyway (**Figure 1**). Similarly, the H5N9 isolate (25WS011-14) displayed a distinct internal gene constellation, clustering largely with the KorD genotype but featuring a PB2 segment related to the KorC genotype, a novel NA segment related to H11N9 viruses detected in Northeast Asia (**Figure 1**). Long terminal branches in the phylogenies, together with the acquisition of novel PB1 and N9 segments in the H5N1 and H5N9 isolate, respectively, suggest that these viruses circulated undetected for approximately three years, during which they underwent reassortment with co-circulating LPAI viruses in wild bird populations (**Supplementary Figure 1**). The identification of HPAI H5N9 virus in South Korea represents a novel subtype introduction in the country’s recorded HPAI history since 2003, highlighting the continued frequent reassortment of clade 2.3.4.4b HPAI viruses with co-circulating LPAI viruses in wild bird populations. The emergence of this HPAI H5N9 virus could alter local viral dynamics by further expanding the genetic diversity of clade 2.3.4.4b viruses. Therefore, close and continuous monitoring of this novel subtype is essential to track its evolution and spread.

ML phylogenetic analysis showed that the 2025 H5N1 and H5N9 isolates clustered closely with recent East Asian H5Nx viruses from Korea, China, Vietnam, and Japan (**Supplementary Figure 1**). The geographical linkage of this introduction was further supported by Bayesian phylogenetic analysis, which showed that the G2c sub-lineage circulating across East Asia since 2022 diverged in mid-2021. Additionally, the H5N1 isolate detected in this study shares a recent common ancestor with a 2023 virus from China (**Figure 2**). These phylogenetic patterns suggest that the newly detected viruses are related to contemporaneous East Asian H5Nx lineages and have arisen through reassortment among viruses co-circulating in the region, rather than through independent emergence, as previously reported (Lee et al., 2017; Sagong et al., 2022; Si et al., 2024; Barman et al., 2025; Si et al., 2025a). The regional connectivity observed across gene segments is consistent with viral movement along the East Asian–Australasian flyway, which links migratory bird populations across these countries (Alkie et al., 2022; Takekawa et al., 2023). However, interpretation of transmission history remains limited by long terminal branches in several segments and by the scarcity of recent genomic data from parts of the region.

Recent East Asian clade 2.3.4.4b HPAI viruses, including the isolates analyzed in this study, share 26 mammalian-adaptation markers across the HA, polymerase complex, M1, and NS segments (**Table 1**). Although the specific combination of substitutions varied among isolates, they shared multiple markers associated with increased virulence, enhanced replication efficiency in mammalian models, along with partial shifts toward α2,6-receptor recognition (Suttie et al., 2019). Although the isolates lacked well-known mammalian-adaptation markers such as PB2-E627K and PB2-D701N, the presence of diverse constellations of other substitutions suggests that their functional significance cannot be fully inferred from sequence data alone. Further experimental studies will be needed to clarify the phenotypic effects of these substitution constellations, and continued genomic surveillance will be important for tracking their distribution and evolution in wild birds and poultry.

In contrast to previous outbreak seasons (2020-2021, 2021–2022, and 2022–2023) (Kim et al., 2024; Seo et al., 2024a; Si et al., 2024), where the index cases were consistently detected in mandarin ducks (*Aix galericulata*), the index case of clade 2.3.4.4b H5Nx HPAI viruses were isolated from a common teal, a highly mobile migratory dabbling duck, on October, 2025, during enhanced active surveillance at the onset of fall migration in South Korea. This small-bodied waterfowl breeds in northern Eurasia and winters across East Asia, utilizing major stopover sites along the East Asian–Australasian flyway (Guillemain and Elmberg, 2014; Takekawa et al., 2023). Its gregarious behavior and preference for shallow wetlands promote frequent reassortment with co-circulating avian influenza viruses, highlighting key role of wild ducks in the dissemination and genetic evolution of HPAI.

Although no HPAI virus had been detected since early summer 2024 in wild birds, sporadic detections at small-scale poultry farms in southern Korea during late June 2024 and again in early autumn 2025 in the northwestern region suggest intermittent reintroduction or low-level viral persistence preceding the migratory season (http://kahis.go.kr). Despite intensive wild bird surveillance, given that HPAI had not been detected in wild birds in South Korea since March 2025 (http://wadis.go.kr), the resumption of wild bird detections underscores the critical role of migratory flyways and wild–poultry interface zones in viral maintenance and dissemination.

In conclusion, the 2025 H5N1 and H5N9 viruses represent continued reassortment and regional gene exchange among East Asian HPAI and LPAI gene pool and were detected as early index cases in common teals during the early 2025–2026 season. Both isolates carried multiple mammalian-adaptation associated substitutions that also present in genetically related East Asian strains. Continued active surveillance, coupled with timely sharing of epidemiological and genomic data, will be essential to enhance understanding of viral dynamics and to strengthen preparedness for newly emerging HPAI lineages.

## Data Availability

The datasets presented in this study can be found in online repositories. The names of the repository/repositories and accession number(s) can be found in the article/**Supplementary Material**.

## Supplementary material

Supplementary Figure 1 Maximum-likelihood (ML) phylogenetic trees of each gene segment of the H5N1 (25WF154-8P) and H5N9 (25WS011-14) highly pathogenic avian influenza viruses isolated in October 2025 in South Korea. Nine ML trees were generated using sequences obtained from the GISAID database, representing **(A)** polymerase basic protein 2, **(B)** polymerase basic protein 1, **(C)** polymerase acidic protein, **(D)** hemagglutinin, **(E)** nucleoprotein, **(F)** neuraminidase (NA) of subtype N1, **(G)** NA of subtype N9, **(H)** matrix protein, and **(I)** non-structural protein. The eight segments of the two viruses identified in this study are highlighted in red, and genotypes of inferred parental segments from Korea (KorD/22G7, KorP, 22G4, and KorC/22G8) are represented in blue. Korean strains are indicated with black bars next to the taxa, accompanied by gray dotted lines. Among them, taxa from ‘21–’22 (light blue), ‘22–’23 (orange), ‘23–’24 (blue), and ‘24–’25 (red) are color-coded accordingly. Bootstrap support values >70 are shown at the corresponding nodes. Heatmaps displayed to the right of each tree illustrate the node support values for the viruses identified in this study, with colors scaled from white (minimum) through light blue (98) to dark pink (maximum, 100).
