# Supplementary figures and images for: Early detection and genetic characterization of clade 2.3.4.4b H5N1 and H5N9 highly pathogenic avian influenza viruses at the onset of fall migration in wild birds during october 2025 in South Korea
Source: Front Cell Infect Microbiol. 2026 Jan 23;16:1755375. doi: 10.3389/fcimb.2026.1755375 (PMC12876187; doi:10.3389/fcimb.2026.1755375)

(A) PB2

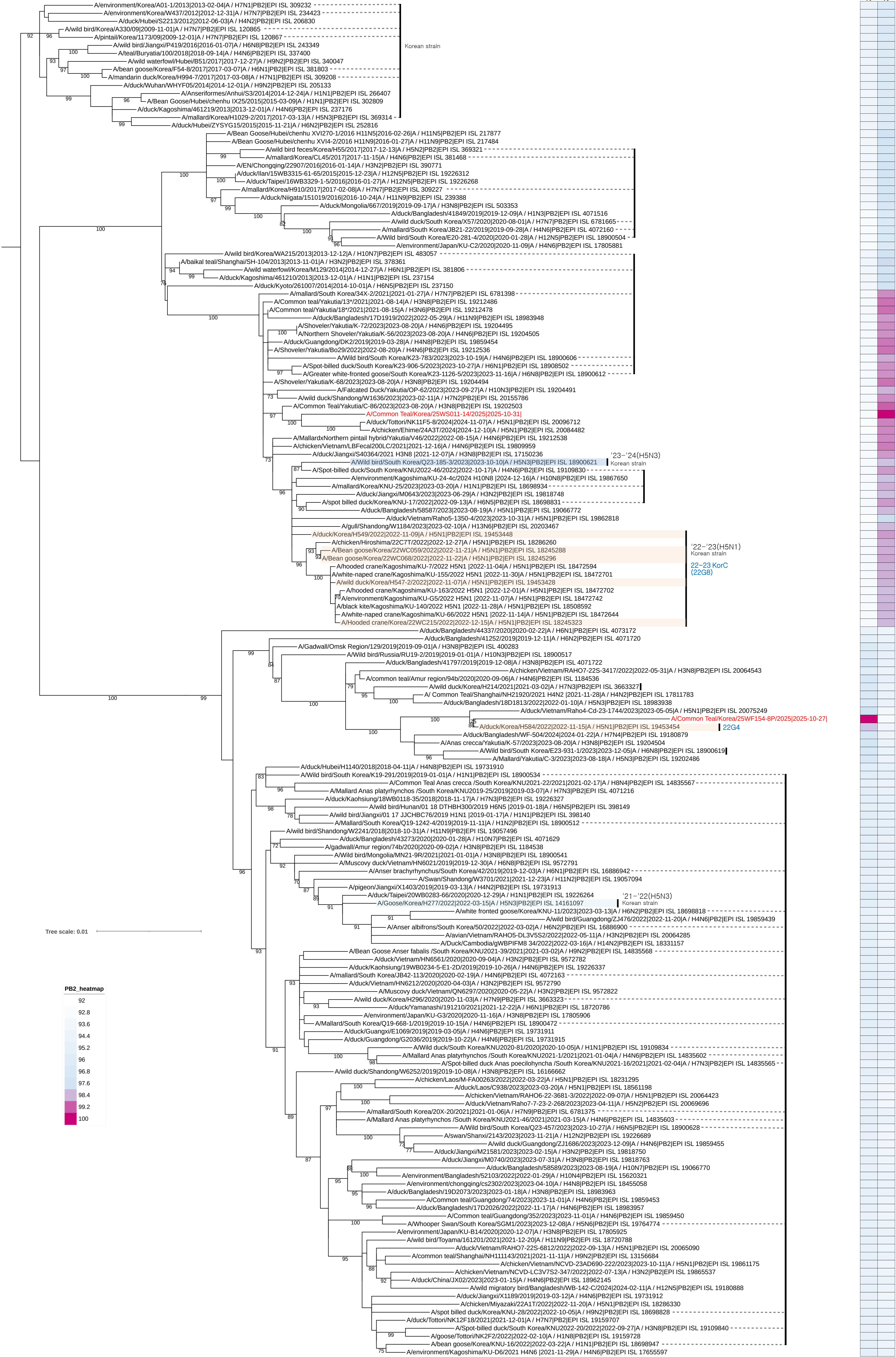

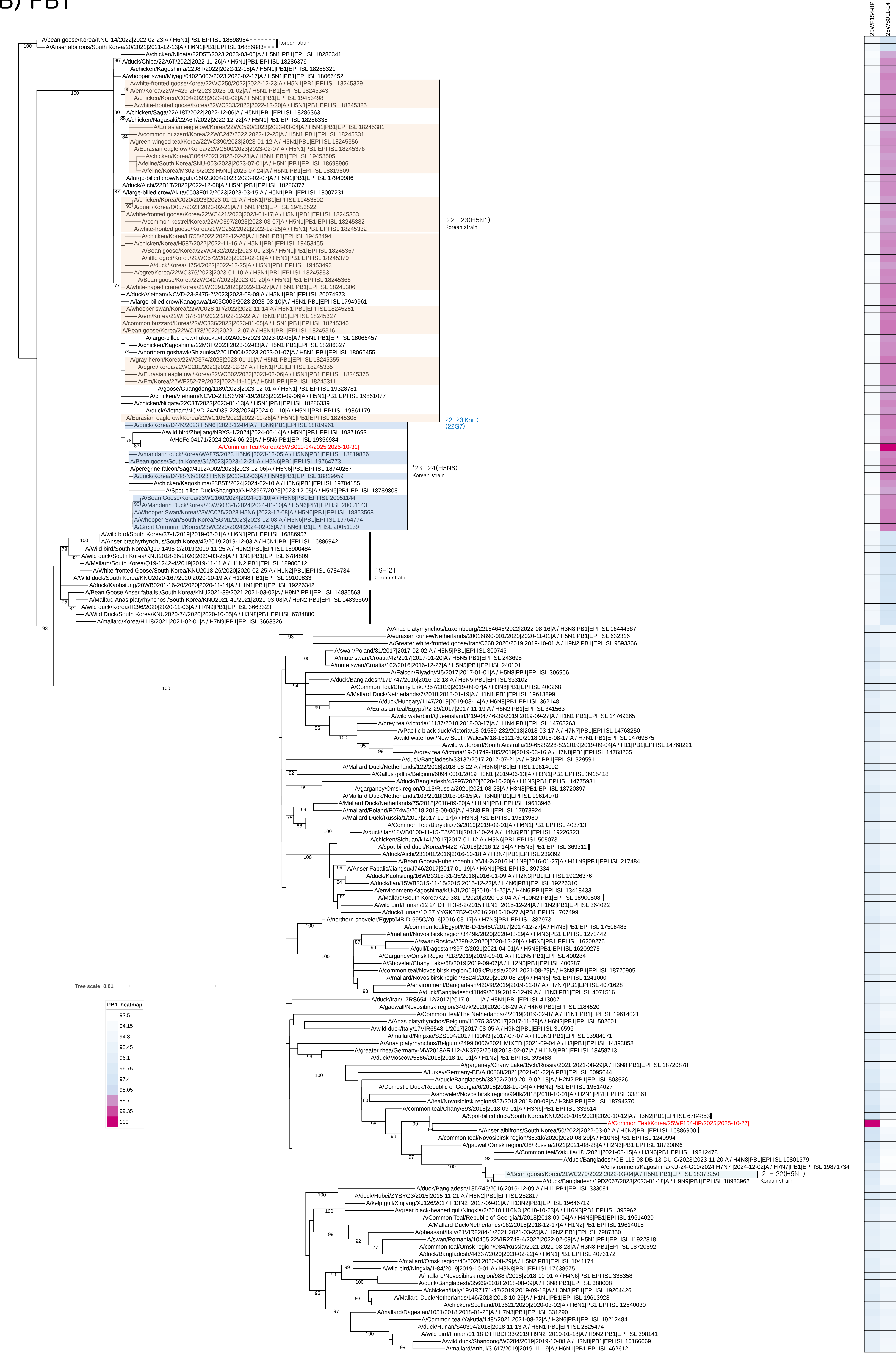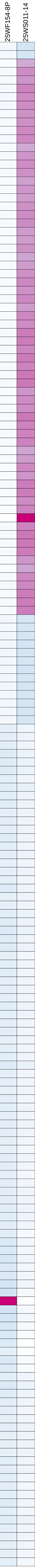

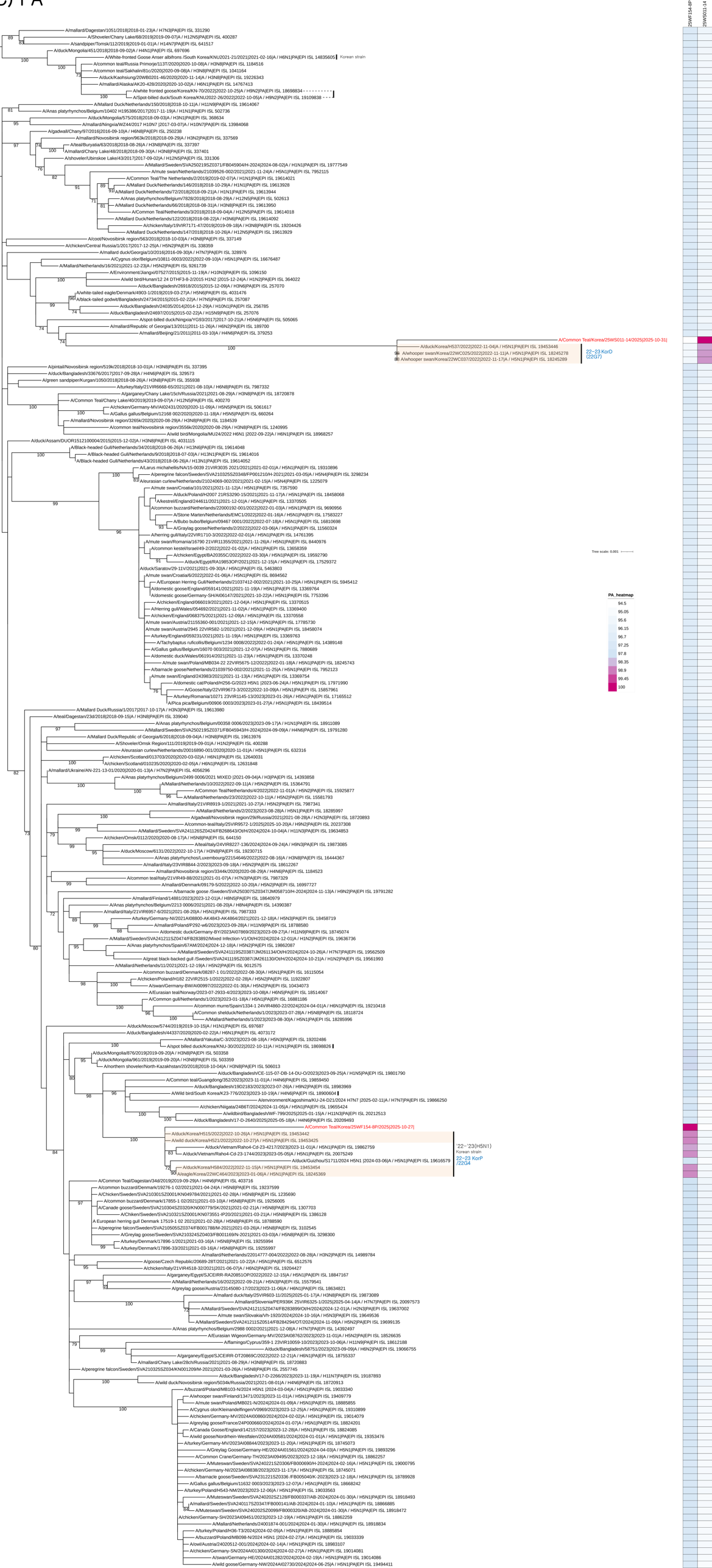

(D) HA

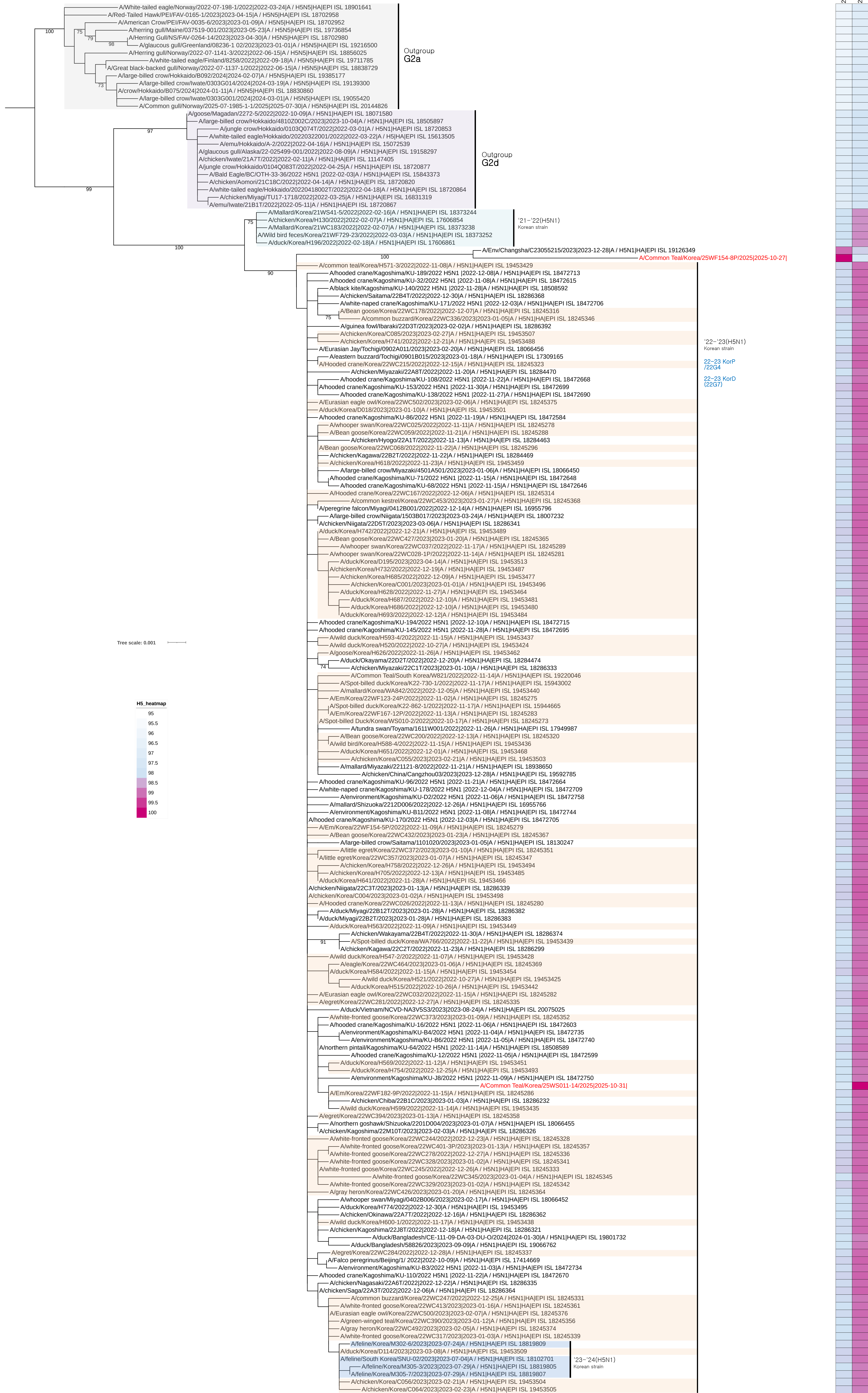

(E) NP

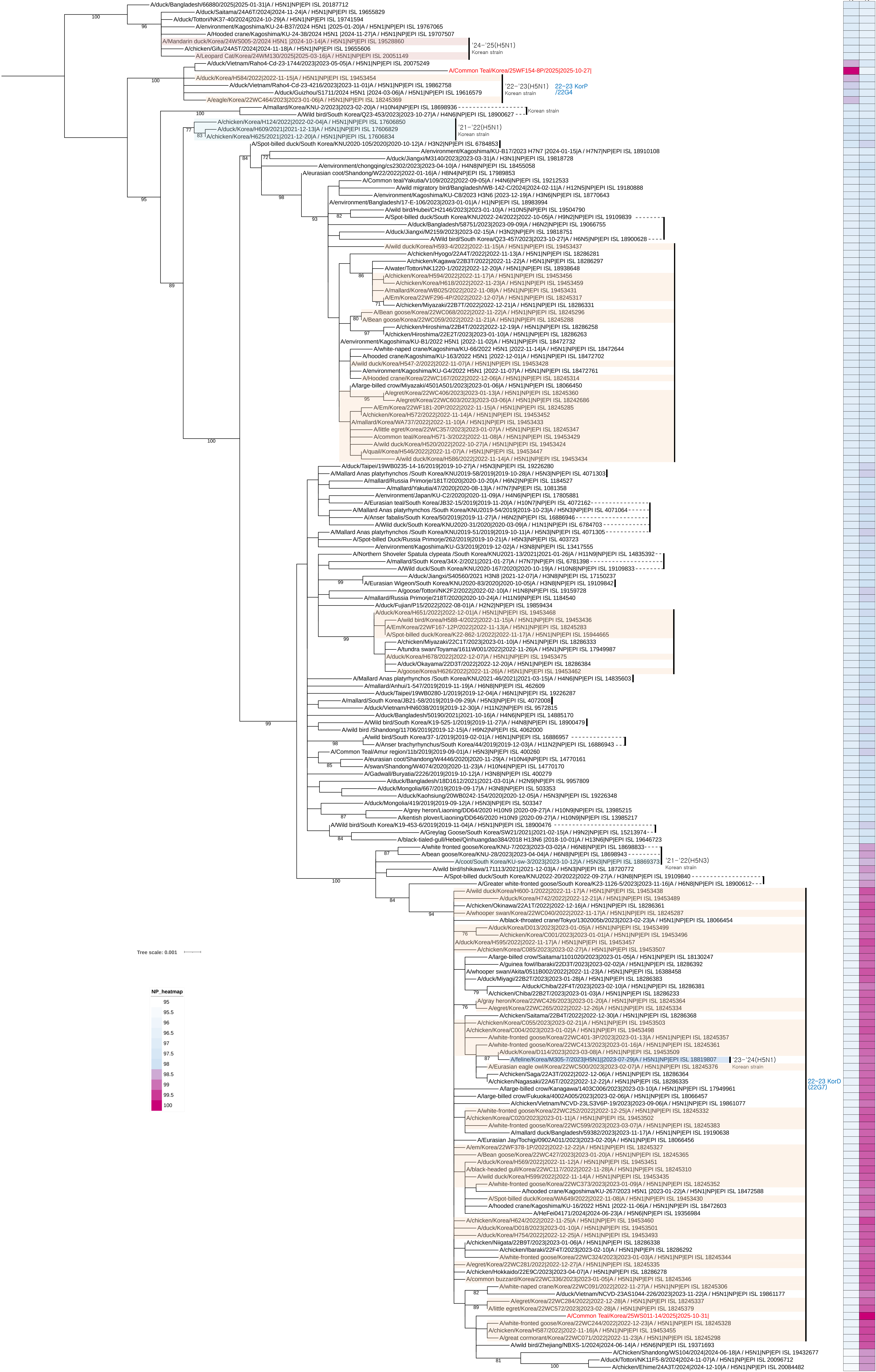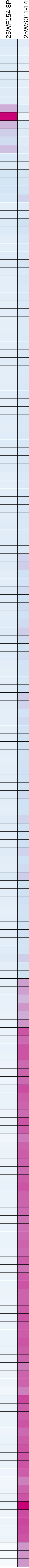

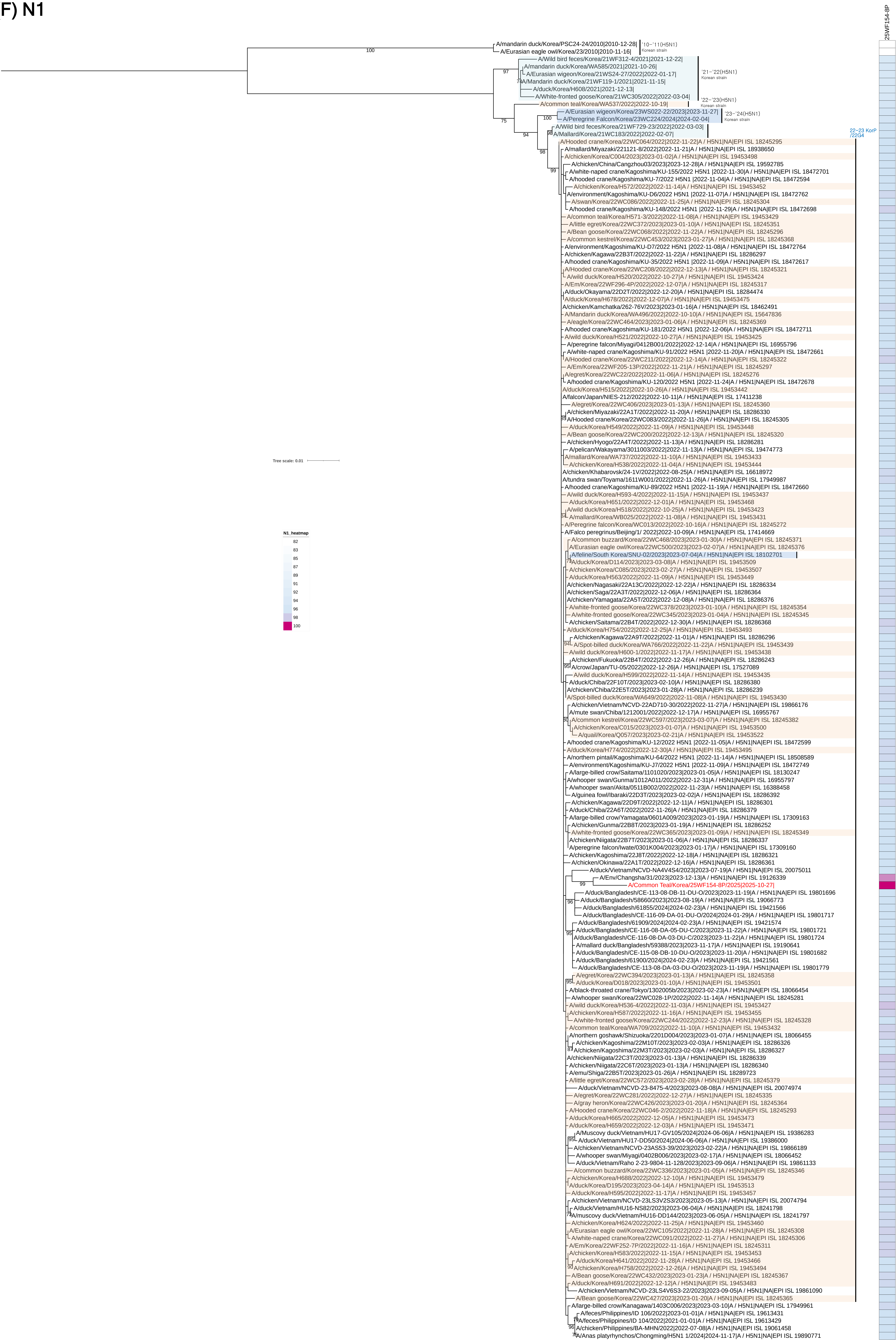

(G) N9

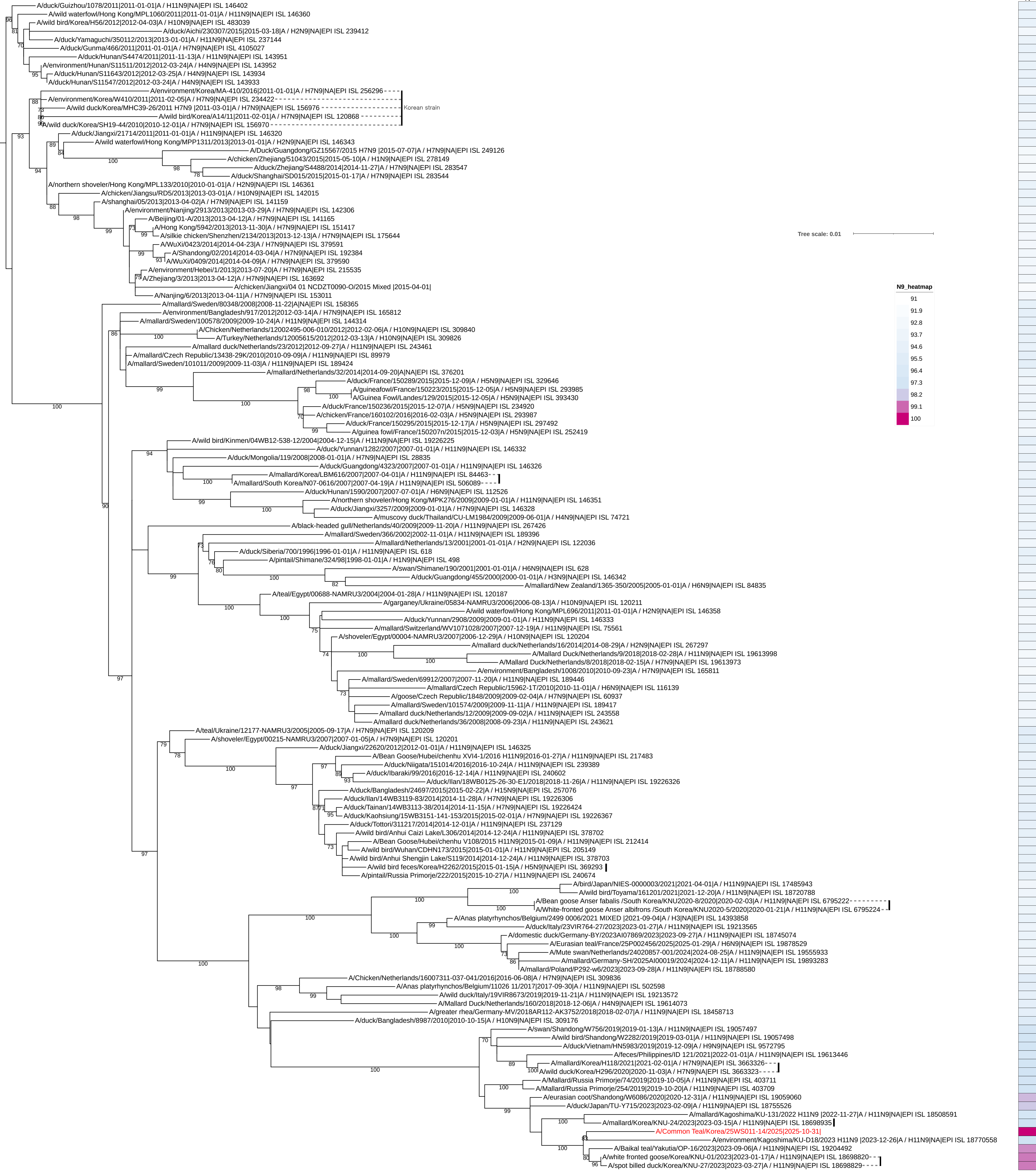

**(H) MP**

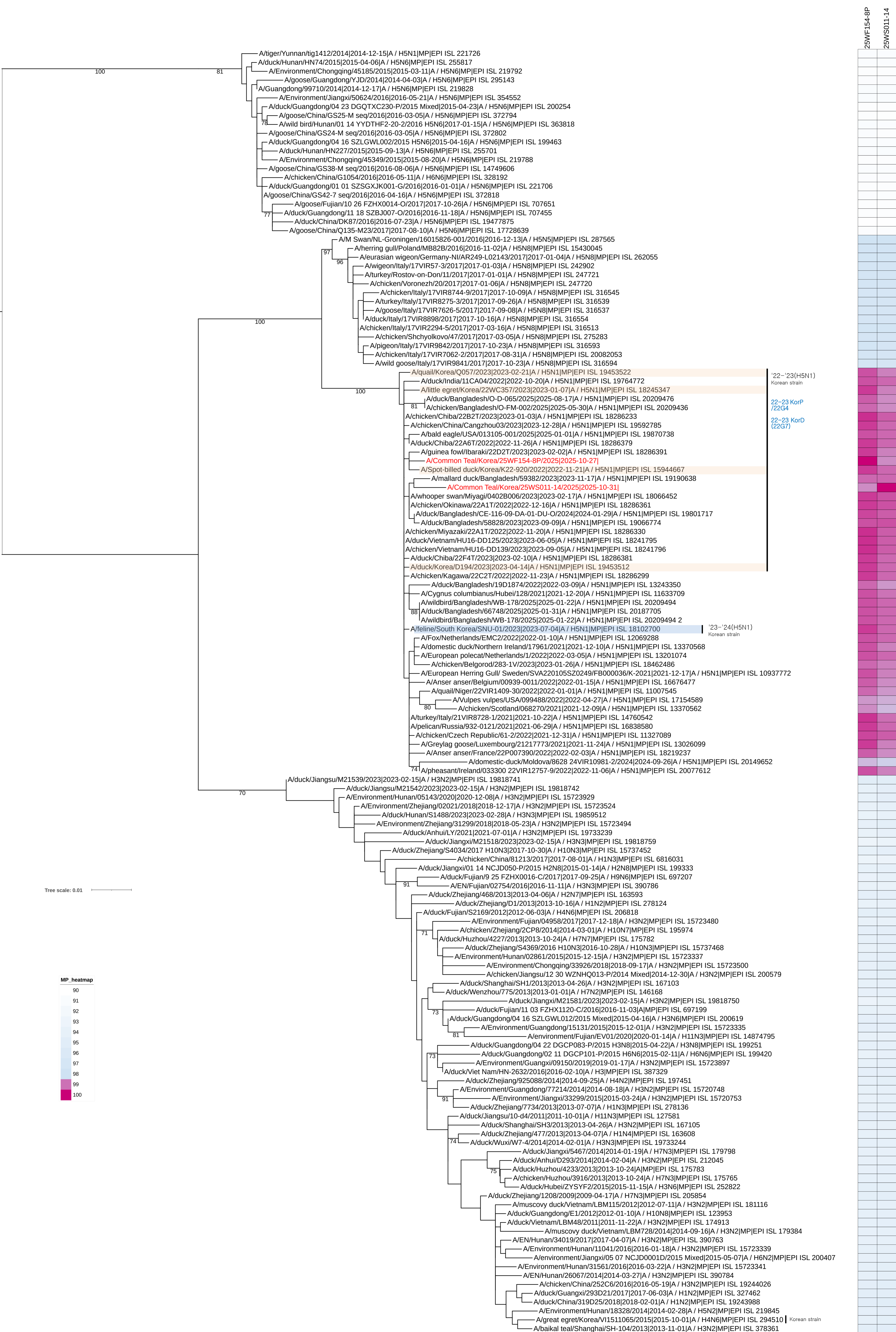

(I) NS

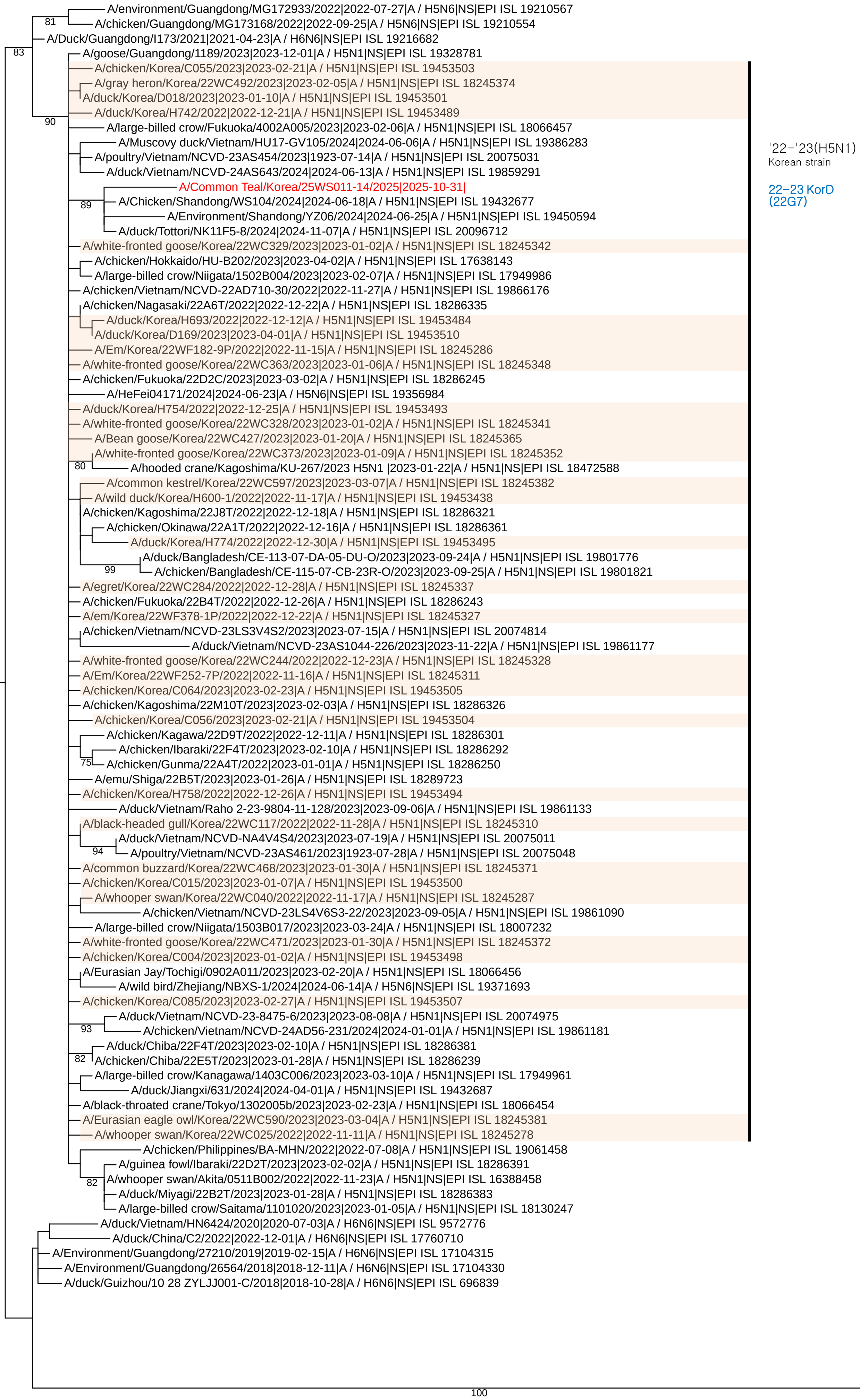

'22-'23(H5N1)  
Korean strain

22-'23 KoR  
(22G7)

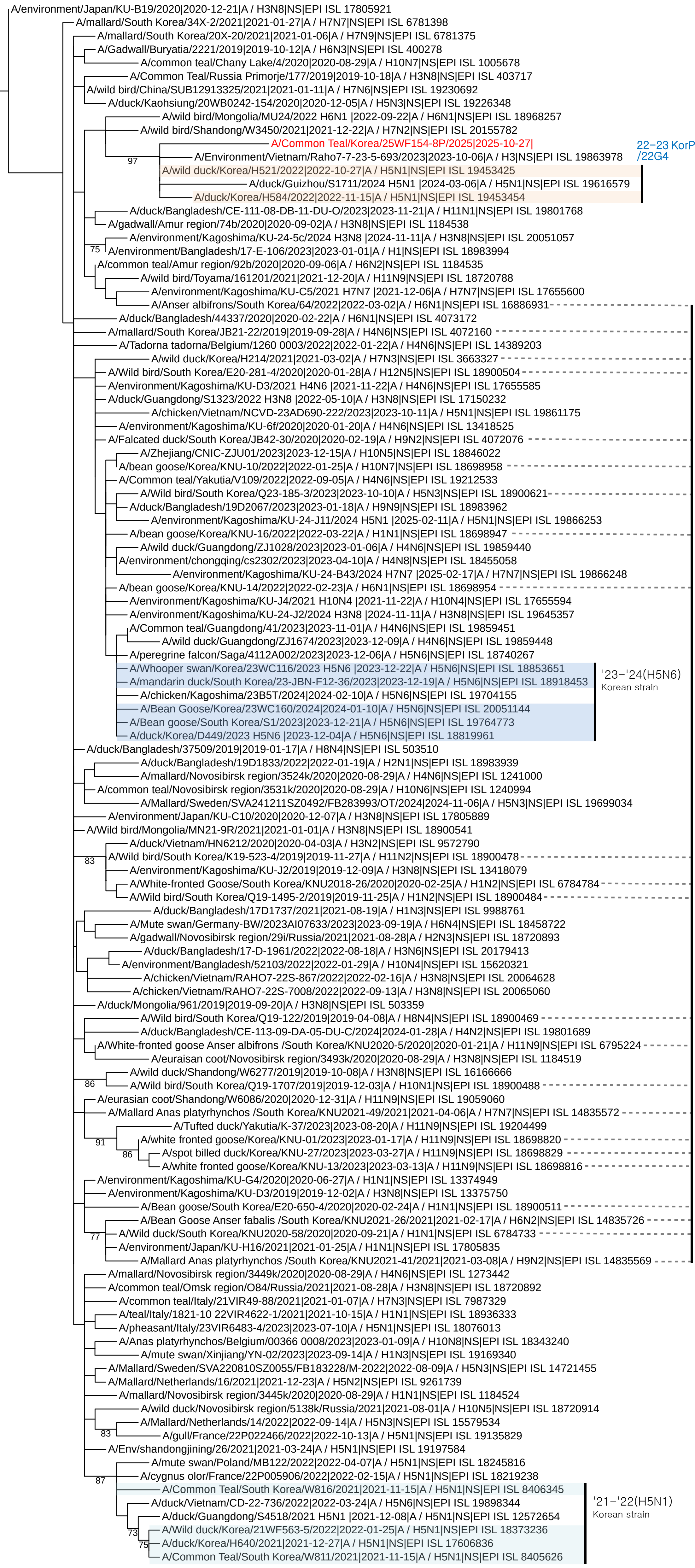

'21-'22(H5N1)  
Korean strain

NS\_heatmap

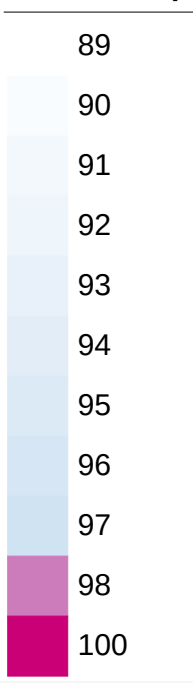

Supplement: Supplementary Figure 1 — Maximum-likelihood (ML) phylogenetic trees of each gene segment of the H5N1 (25WF154-8P) and H5N9 (25WS011-14) highly pathogenic avian influenza viruses isolated in October 2025 in South Korea. Nine ML trees were generated using sequences obtained from the GISAID database, representing (A) polymerase basic protein 2, (B) polymerase basic protein 1, (C) polymerase acidic protein, (D) hemagglutinin, (E) nucleoprotein, (F) neuraminidase (NA) of subtype N1, (G) NA of subtype N9, (H) matrix protein, and (I) non-structural protein. The eight segments of the two viruses identified in this study are highlighted in red, and genotypes of inferred parental segments from Korea (KorD/22G7, KorP, 22G4, and KorC/22G8) are represented in blue. Korean strains are indicated with black bars next to the taxa, accompanied by gray dotted lines. Among them, taxa from ‘21–’22 (light blue), ‘22–’23 (orange), ‘23–’24 (blue), and ‘24–’25 (red) are color-coded accordingly. Bootstrap support values >70 are shown at the corresponding nodes. Heatmaps displayed to the right of each tree illustrate the node support values for the viruses identified in this study, with colors scaled from white (minimum) through light blue (98) to dark pink (maximum, 100). [file DataSheet1.pdf]
